# A Mediterranean coastal database for assessing the impacts of sea-level rise and associated hazards

**DOI:** 10.1038/sdata.2018.44

**Published:** 2018-03-27

**Authors:** Claudia Wolff, Athanasios T. Vafeidis, Sanne Muis, Daniel Lincke, Alessio Satta, Piero Lionello, Jose A. Jimenez, Dario Conte, Jochen Hinkel

**Affiliations:** 1 Coastal Risks and Sea-Level Rise Research Group, Christian-Albrechts-University Kiel, Department of Geography, 24098 Kiel, Germany; 2 Institute for Environmental Studies (IVM), Vrije Universiteit Amsterdam, 1081 HV Amsterdam, Netherlands; 3 Global Climate Forum, 10178 Berlin, Germany; 4 Mediterranean Sea and Coast Foundation, 09123 Cagliari, Italy; 5 CMCC, Euro Mediterranean Center on Climate Change, 73100 Lecce, Italy; 6 DiSTeBA, University of Salento, 73100 Lecce, Italy; 7 Laboratori d’Enginyeria Marítima, Universitat Politecnica de Catalunya – BarcelonaTech, 08034 Barcelona, Spain; 8 Berlin Workshop in Institutional Analysis of Social-Ecological Systems (WINS), Humboldt-University, 10099 Berlin, Germany

**Keywords:** Natural hazards, Environmental chemistry

## Abstract

We have developed a new coastal database for the Mediterranean basin that is intended for coastal impact and adaptation assessment to sea-level rise and associated hazards on a regional scale. The data structure of the database relies on a linear representation of the coast with associated spatial assessment units. Using information on coastal morphology, human settlements and administrative boundaries, we have divided the Mediterranean coast into 13 900 coastal assessment units. To these units we have spatially attributed 160 parameters on the characteristics of the natural and socio-economic subsystems, such as extreme sea levels, vertical land movement and number of people exposed to sea-level rise and extreme sea levels. The database contains information on current conditions and on plausible future changes that are essential drivers for future impacts, such as sea-level rise rates and socio-economic development. Besides its intended use in risk and impact assessment, we anticipate that the Mediterranean Coastal Database (MCD) constitutes a useful source of information for a wide range of coastal applications.

## Background & Summary

The Mediterranean basin is characterized by a squeezed coastal area with a high concentration of people and assets and by rapid demographic, social, economic as well as environmental change^1,2^. Between 1960 and 2010, the population of the Mediterranean has doubled from 240 million to 480 million^3^ and the urban population has increased by 20% (ref. 2). This human pressure is further amplified by international tourism. Around one third of the global tourist arrivals in 2011 have been registered in Mediterranean countries, predominantly along the coast. The number of arrivals is expected to increase further, and could reach 637 million per year by 2025 (ref. 3). The Mediterranean coastal zone is not only increasingly under pressure from local human activities, but also subject to future global environmental change. In particular, sea-level rise and associated hazards^4^ are expected to have significant impacts in Mediterranean nations during the 21^st^ century^5–7^.

To address these pressures and to underpin future coastal management and adaptation policies, such as those included in the Integrated Coastal Zone Management (ICZM) Protocol of the Barcelona Convention^8^, policy makers and coastal administrations in Mediterranean nations require impact and vulnerability assessments. Such integrated assessments are cross-sectorial studies that require information from various fields and disciplines. A prerequisite for coastal impact assessment and for the planning of appropriate future interventions is the availability of consistent information on the physical, ecological and socio-economic characteristics of the Mediterranean coastal zone. Despite an increasing demand of decision-makers, planners and coastal researchers from various disciplines for such consistent scientific data^9,10^ there is currently no source of readily available information for the 22 countries that surround the Mediterranean basin. Due to the lack of such data only a limited number of studies exist that have analyzed the impacts of sea-level rise for the entire region^10,11^. Collecting and organising such data is a challenging task as consistent information on socio-economic and physical characteristics, both on current conditions as well as on future developments, of the coast is needed.

The developed MCD aims to meet these needs through the provision of an open access spatial database, that provides consistent information (in terms of format, resolution, quality, accuracy) for the entire region and that is based on a lean data model. The coastal database contains 160 parameters that characterize the natural and socio-economic systems of the Mediterranean coast. It relies on the structure that was originally designed for the Dynamic Interactive Vulnerability Assessment (DIVA) modeling framework^12–14^, following the concepts described in reference^15^ and reference^16^ However, we have downscaled and extended these approaches by introducing spatial coastal assessment units that capture the spatial structure of population, assets and land exposed to coastal hazards. Further we have enhanced the transparency of the process of attribution of spatial data to the segments and coastal assessment units in order to make the database more user friendly. The developed coastal database is intended for use in regional scale analyses and provides a robust basis for all types of comparative coastal studies to future change as it allows results to be comparable across the entire region.

In this data descriptor we describe the generation of coastal segments and associated spatial coastal assessment units; the methods that we have used to attribute around 160 different parameters on current and future conditions in various formats to the coastal units; and the development of consistent data for the Mediterranean coasts.

## Methods

### Coastal data model

Finding a data model to represent coastal space for vulnerability, impact and adaptation assessment is not a straightforward task as coasts are highly dynamic and complex in terms of process interactions^17^. This introduces challenges when trying to depict this system into a format that allows spatial information to be stored into a database. A linear representation of the coastal zone has often been used in coastal studies due to the common perception of the coast as a linear boundary between the sea and the land^18^. The main advantage of a linear data model is its computational efficiency, which is essential for being able to conduct the large number of model runs needed in impact and adaptation assessments as a result of the large number of plausible sea-level rise, socio-economic or adaptation scenarios available. However, there is no direct way to attribute spatial data, such as the number of people living in the low-elevation coastal zone or landuse covering the coastal space, to a linear feature without losing spatial information. Therefore, a main disadvantage of a linear data model is that the explicit spatial structure of the system is lost^19^. The alternative data model frequently applied in order to preserve coastal spatial information is a grid. The disadvantage of this model is, however, that data volumes are large and computationally expensive for use in impact and adaptation assessments at broader geographic scales.

**Figure 1:**
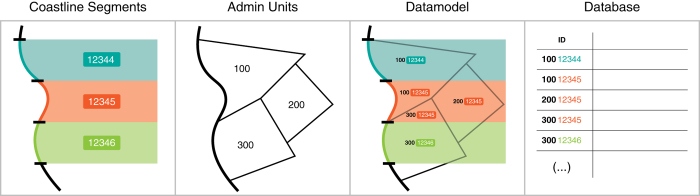
Workflow of the data model generation for the Mediterranean coastal database.

To address this challenge, we have created a data structure that combines the linear representation of the coast with spatial coastal assessment units that extend inland (Figure 1). Thus, we combined the advantages of a linear data model with a spatial representation of the coastal zone. To create such a data model, the first step was to divide the coast into homogenous segments in terms of impacts, vulnerability and adaptation to sea-level rise. For each segment, we then created a set of spatial coastal assessment units according to administrative boundaries and extent of land up to 20 m of elevation above mean sea level. Our aim was to generate assessment units that will respond uniformly to sea-level rise and can be treated as single units for the purpose of adaptation planning in the future. In the following section, we describe the data model and present the computational data processing that was undertaken for populating the database.

### Coastal segments

**Figure 2:**
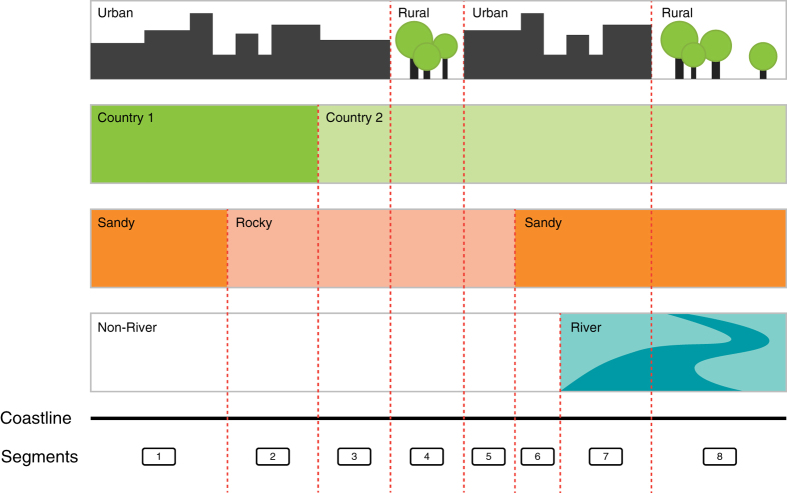
Schematic segmentation procedure for the Mediterranean coastal database.

Following the concept of reference^15^ and reference^20^, we generated coastal segments of variable length, each segment representing parts of the coast with a uniform vulnerability to sea-level rise at a regional scale. As differences in vulnerability to sea-level rise are driven by variations in both socio-economic as well as geomorphic/physical characteristics of the coastal zone^16^, we used four parameters covering both these domains in the segmentation decision. Every time one of the four parameters changed in value, a new segment was started (Figure 2).

We employed the coastline of the Global Administrative Areas^21^ database level 01 as the base layer for the segmentation for the Mediterranean countries (Figure 2) and corrected artefacts related to the format (e.g., “pixelization” of coastline) using a smoothing algorithm (polynomial approximation) and a tolerance of 100 meters.

The first social system parameter included in the segmentation were (1) *administrative boundaries.* This parameter was included, because society’s capacity to respond to sea-level rise differs across jurisdictions. In this study, we used the GADM level 02 dataset (see Table 1).

The second social system parameter we used in the segmentation was the (2) *distribution of assets and people* distinguishing the coast into two classes, (a) urban and (b) rural. This parameter is relevant for the segmentation, because population and asset density influence vulnerability by both determining the exposure to sea-level rise and storm surges, as well as by influencing society’s’ capacity to adapt^13^. We classified the coast using satellite imagery and photos from Google Earth and the Moderate Resolution Imaging Spectroradiometer (MODIS) global map of urban extent dataset with a spatial resolution of 500 m^22^. Classification decisions were based on visual interpretation of Google imagery and on the MODIS data, where urban areas are defined as places with predominantly built environment. That includes all non-vegetative, human-constructed elements, such as buildings, roads, runways and are greater than 1km^2^ (ref. 22). All pixels with a coverage greater than or equal to 50% built environment according to the MODIS dataset^22^ were classified as urban. As we were particularly interested in all human settlements that are lying directly on the coast, we refined the classification using Google Earth in order to also include smaller human settlements. Therefore, settlements with a maximum distance of 300 m to the shoreline and a minimum extent of 300 m x 300 m, predominantly covered by residential buildings, were defined as urban. Harbours were excluded from the urban classification, as they will require specific adaptation measures in the future.

The first geomorphological parameter we considered in the segmentation was the (3) *coastal material*. The material of the coast has a significant impact on the large-scale response to sea-level rise^16^. One of the major impacts of sea-level rise is long-term erosion and land loss due to permanent inundation^12^. For instance, sandy beaches will respond differently than rocky coasts to a rising sea level. We created a typology of four different geomorphic classes that respond differently to rising sea level and will require different adaptation measures. For the Mediterranean, no such dataset on coastal morphology and geological characteristics was available at that time. Four different classes, namely (a) sand, (b) unerodible, (c) mud and (d) rock with pocket beaches have been classified based on visual interpretation of Google Earth imagery and location-tagged photographs from the web-service Panoramio which offers geographically tagged photographs from users^23^. A similar method has been used in reference^20^ and reference^23^.

The second geomorphological parameter that we included in the segmentation process is (4) *river mouths.* This parameter was included because deltas and estuaries are among the most vulnerable coastal geomorphic features to sea-level rise^24^. We classified 47 river mouths for the Mediterranean region based on Google Earth imagery.

Finally, those layers with the four different parameters were combined to create the coastline segmentation for the Mediterranean. Overall, there are 11,975 segments with an average length of 4.5 km.

### Spatial assessment units

The spatial assessment units expanding inland were created by generating inland buffer zones for every coastline segment and overlaying them with elevation data and the administrative boundaries (resolution: 3 arc second). We only account for the low-lying part of the coastal zone that is hydrologically connected to the sea and lies above 20 m from mean sea level. The low-lying part of the coast is particularly at risk due to higher extreme sea levels in the future^25,26^. We decided to extend the LECZ (Low Elevation Coastal Zone=Area below 10m^27–29^) up to 20 m in order to account for all plausible scenarios of changes in mean sea level and associated hazards, including high-end scenarios, as well as to allow exploring different adaptation strategies such as coastal retreat. Every coastal assessment unit was linked to a coastal segment with a unique identifier code. The coastal segment unique identifier code consists of eight digits. The first three represent the administrative unit and the last five digits represent the coastline segment (Figure 1). The generated coastal data model forms the basis for the subsequent compilation of the database.

### Extreme sea levels and waves

The MCD includes two extreme sea level datasets. The first dataset included is derived from the Global Tide and Surge Reanalysis (GTSR) dataset. GTSR is the first near-coast global reanalysis of storm surges and is based on global hydrodynamic modelling combined with meteorological forcing from ERA-Interim (1979–2014). A Gumbel extreme value distribution was fitted to the annual maximum to derive extreme sea levels for various return periods. GTSR generally provides extreme sea levels for the centroids of the coastal segments of the global database used in the DIVA model. However, for the MCD we increased the spatial resolution and saved the outputs for the 11 975 centroids of the Mediterranean coastline segments (GTSR-MED). The general methodology is described in detail in reference^30^.

The second dataset that we included in the MCD is the DINAS-COAST Extreme Sea Levels (DCESL). This dataset has been the first global extreme water level dataset and was developed with the use of a simple empirical model described in detail in refs 31,32.

Besides the data on extreme sea levels, we have also included information on mean wave heights for the period 1971–2000 for the Mediterranean basin. The wave data have been computed using the wave model WAM^33^ at a resolution of 0.25 degrees, resolving the spectrum using 12 directions and 25 frequencies. The wind meteorological forcing was generated using the hourly meteorological fields produced by the regional climate model COSMO-CLM at a resolution of 0.12 degrees. The model framework has been validated by reference^34^ and reference^35^. These mean wave heights should be considered representative of offshore conditions, before depth induced wave breaking and interaction with the bottom in the near shore zone occur.

### Computational data processing

The database was developed with the use of ArcPy, which is a site-package that builds on the ArcGIS scripting module. It enables users to perform geographic spatial data analysis, data conversion and data management with the programming language Python. One main advantage is that every Python script constitutes a precise documentation of the computational data processing that was conducted. The input spatial data were available in various formats depending on the information that they represented. Therefore, we used different methods to attribute the data to the coastline segments and spatial assessment units.

Data processing differed according to two characteristics of the input data, namely: (1) whether the data were originally in vector or raster format; and (2) whether we attributed them to the coastline segment or to the assessment units (e.g. data representing information that extends several kilometres inland). For instance, the extreme sea level data were available in a vector (point) format representing extreme sea levels for different return periods directly at the coast. We spatially joined the nearest extreme sea level data point to the centroid of every segment in order to have a common attribution approach. Every coastal assessment unit obtained the extreme sea level information of the corresponding coastline segment (Figure 1). Another example for the attribution of raster data to the coastal assessment units is the information about the distribution of people in the coastal zone. For this purpose, a gridded population dataset was combined with gridded elevation data in order to calculate the number of people below a certain elevation (in 1 m increments, up to 20 m). Then we calculated the zonal statistics for every coastal assessment unit in order to get the number of people per elevation increment. A detailed documentation for every step employed for attributing the different parameters to the coastal units can be found in the Python scripts (Table 1 and Table 2).

The database consists of various parameters about current conditions of the coastal zone. In addition, the database provides information on plausible future changes that will drive future impacts, such as sea-level rise or socio-economic development scenarios. Table 1 and Table 2 summarizes all parameters that are included in the database; their source; a short description and the name of the python code where the detailed computational data processing steps are documented. With the exception for the extreme sea level datasets and the wave data, we only used datasets that are publically available.

### Code availability

The python code to populate the Mediterranean coastal database is available to download in the figshare repository (Data Citation 1). The code consists of ArcPy commands, which can be used if an ArcGIS for Desktop license is installed. Each script is internally documented with explanation of the different data processing steps. The internal documentation of the scripts should be used in combination with this manuscript.

## Data Records

The developed Mediterranean coastal database described in this article is publicly and freely available through the figshare repository. We have included csv files with all the information on a segment (MCD – coastal segment level) and assessment unit level (MCD – coastal assessment unit level) into the repository. Furthermore, we provide the coastal segments and administrative boundaries in a shapefile format as well as the coastal assessment unit in a tiff format in the repository. The database will be updated and expanded as new and improved data become available.

## Technical Validation

The database presented here has been created using a number of publically available datasets, which are thoroughly documented and described in reports or scientific articles (Table 1 and Table 2). Thus, these datasets have undergone rigorous quality controls and/or validation. In addition, for those parameters where consistent information for the entire basin did not exist, new datasets were generated. Technical validation therefore has focused on the evaluation of these new datasets (i.e. the coastal material, GTSR-MED extreme sea levels); and on the correct attribution of data to the assessment units.

### Geomorphological classification

The geomorphological classification dataset was compared to the recently compiled geomorphological dataset of the Mediterranean Sea and Coast Foundation (MEDSEA, 2017) which was developed independently, using a similar methodological approach. Further validation for some of the Mediterranean countries, where this was possible, was undertaken based on expert judgement and on national digital datasets of coastal morphology.

Comparing different coastal classification datasets can be a challenging task as the number and type of classes used can differ substantially, depending on factors such as scale or user requirements^36,p.267^. The MEDSEA data included similar classes to our dataset, namely: (1) sandy beach and beach with uncertain grain size; (2) river, deltas; estuaries and soft sedimentary strands; (3) artificial structures and artificial frontage; (4) soft rock shores; and (5) hard rock shores (see Table 3). 23% of the coast were classified as sandy in both datasets. The MCD class ‘unerodible’ includes rocky coasts and artificial structures. When comparing the MCD class (2) and the MEDSEA classes (3) and (5), the datasets show good agreement, accounting for 46 (MCD) and 40 (MEDSEA) percent of the total coastline. The remaining classes (“rocky with pocket beaches” and “soft rock shores” from MCD and MEDSEA respectively) are not directly comparable. Overall, the two datasets are in agreement for around 70 percent of the Mediterranean coast. The spatial patterns of coastal types are similar in the southern and eastern Mediterranean basin. In the north western part of the basin differences in the classification are visible as the MEDSEA dataset indicates a higher extent of hard and soft rock shores. However, this difference is primarily due to the difference in the definition of the classes for the two classification schemes.

Local experts from Spain, Greece and Croatia undertook additional checks, based on visual inspections and on national or local datasets. For Spain, there was agreement for 75% of the coast. For the remaining 25%, we implemented changes based on the expert suggestions and additional checks with Google Earth. For Croatia, we compared our dataset to a national spatial dataset on the distribution of sandy beaches provided by the Ministry of Environmental and Nature Protection and found that all sandy beaches were included. Further qualitative tests based on expert judgement and visual assessment were carried out for Greece for approximately 100 randomly selected segments, indicating an agreement for approximately 85% of the inspected segments. Accordingly, discrepancies were checked and corrected based on Google Earth and expert suggestions. Finally, further comparisons were carried out for the districts of Lazio and Emilia-Romagna with available classifications used in previous analyses^20^.

It must be noted that despite the extensive evaluation of the geomorphological classification and the agreement with all employed data sources, some errors may still exist. These errors can result from numerous factors, such as differences in the quality of Google Earth imagery for the entire region; differences in scale; errors in the location of Panoramio photographs; coverage of location-tagged photographs varying considerably between the Northern and Southern part of the basin; errors in classification; or differences due to subjectivity in class definition. Nevertheless, the dataset will continue to be updated as new data become available. We are currently exploring options for extending the validation using crowd sourced data from the project Coastwards (http://coastwards.org/) in order improve our current classification.

### Extreme sea level datasets

Here we compare the two datasets of extreme sea levels against observations. First, we evaluate GTSR-MED and DCESL which include tides and surges.

**Figure 3:**
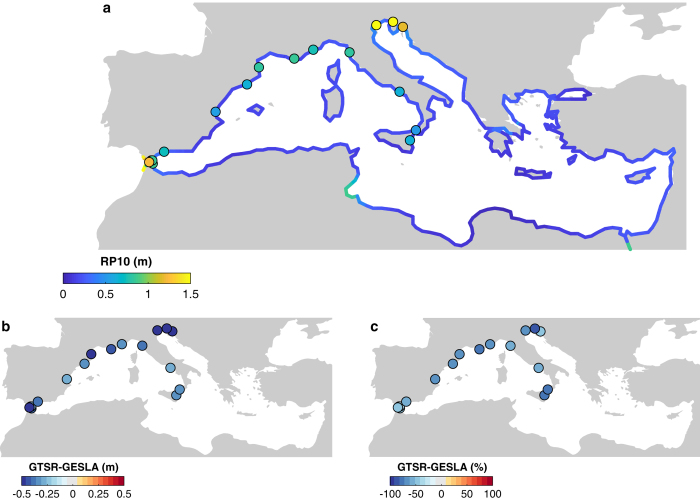
Performance of the GTSR-MED extreme sea levels for the Mediterranean Sea for the 10-year return period. Observed extreme sea levels are taken from the GESLA dataset. (**a**) GTSR-MED and GESLA extreme sea levels. (**b**) The difference of GTSR-MED with GESLA in m. (**c**) Relative differences of GTSR-MED and GESLA.

Figure 3a shows a comparison of the modelled and observed extreme sea level with a 10-year return period for the GTSR dataset. Extreme sea levels are generally in the range of 0.15 m and 1.24 m. Off the coast of Tunis, the Strait of Gibraltar, and near Venice extreme sea levels are relatively high. This is corresponding with the relatively high tidal range in these areas. For example, the Gulf of Gabes off the coast of Tunisia has a tidal range of nearly two meters, while tides are generally small in other parts of the basin. Reference^30^ evaluated the GTSR extreme sea levels against observed sea levels from the archive of the University of Hawaii (http://uhslc.soest.hawaii.edu). However, the set of tide gauges used for the global validation has a very limited number of tide gauges available in the Mediterranean basin. Therefore, we performed additional validation using the GESLA-2 dataset (http://gesla.org), which includes data from many more tide gauges^37^. Here we use the estimates of the return periods of extreme sea levels from reference^38^. They processed the raw data and fitted a Gumbel distribution to the annual maxima using all stations that contain at least 20 complete years that is less than 25% missing data. For the Mediterranean region, this resulted in 17 observation stations. To evaluate the performance of the GTSR-MED extreme sea levels, we calculate the mean bias and the mean absolute error between the modelled and observed extremes. The modelled extreme sea levels are generally characterized by a negative bias. For a 10-year return period the mean bias is −0.21 m (s.d. 0.20 m), whereas for a 100-year return period the mean bias is −0.34 m (s.d 0.41 m). As extreme sea level are generally below 1.5 m, the relative differences exceeds 25% for a number of locations. This is depicted in Figure 3a-c. The relatively strong negative bias may be due the fact that in semi-enclosed basins, such as the Mediterranean Sea, extreme sea levels are largely controlled by local conditions and mesoscale dynamics. Hence, the representation of the global bathymetry and the resolution of the global tide and surge model may not be sufficient in this region. Moreover, in areas with a complex orography, such as the Adriatic Sea, global climate reanalysis data can have difficulties in reproducing local winds^39^. Unfortunately, almost all tide gauges are located in Portugal, Spain and Italy. Hence, the eastern and the southern part of the Mediterranean Sea are under–represented. Therefore we were not able to assess the performance of the model for the entire basin.

The DCESL data have been validated by reference^32^. The study concluded that the return periods of DCESL are generally too high, compared to observed return periods from GESLA-2. Reference^31^ compared the GTSR and DCESL against UHSCL observations. They found that both GTSR and DCESL capture the spatial variability of extremes. However, DCESL generally overestimates the extreme sea levels, whereas GTSR generally underestimates the extreme sea levels, but with smaller errors compared to observations.

However, this comparison included only few stations in the Mediterranean. The comparison of GTSR and DCESL by reference^38^ is based on the GESLA-2 dataset and includes more stations. It shows that DCESL overestimates the 100-year return period values by more than 0.5 m. If we compare the DCESL return periods against the GESLA-2 stations used for the GTSR-MED validation (see above), the mean bias is 0.16 m (s.d. 0.43 m), 0.17 m (s.d. 0.59 m), and 0.20 m (s.d. 0.76 m), respectively for the 10-, 100-, and 1000-year return periods. For specific stations the bias is up to 1.5 m, whereas for other stations the bias of DCESL is less than a few centimetres. Hence, although the performance is variable, DCESL generally also overestimates extreme sea levels in the Mediterranean basin. Again, there is no information to assess DCESL for the eastern and the southern part of the Mediterranean Sea.

We would like to highlight that due to the limited numbers of observations it is difficult to assess the overall performance of the extreme sea level datasets. However, we recommend users that would like to perform an analysis on a regional scale to use the GTSR-MED extreme sea levels as the standard deviation (observed vs modelled) is smaller. Users that are interested in a specific location may be more interested in the DCSEL dataset as the mean bias is smaller than for the GTSR-MED dataset, which indicates that the modelled extreme sea levels may be closer to the observed values in some locations. Users interested in coastal flood risk assessment to extreme sea levels should be aware that both datasets do not include waves. Omitting waves could lead to an underestimation of potential impacts (see reference^40^).

### Data attribution

The attribution of data to the coastal units involved several processing steps, which varied depending on the type of dataset (as documented in the python scripts, see methods section). Every parameter of the database was then checked manually in a Geographic Information System, by at least two different users, to ensure correct attribution. This validation process was introduced to ensure internal consistency and, to identify and correct errors or mismatches in the database compilation process.

## Usage Notes

We envisage that academics, managers and planners will use the developed database for coastal applications. We emphasize that the database is designed for regional-scale applications. Caution is required when using the database for local applications.

## Additional information

**How to cite this article**: Wolff C. *et al.* A Mediterranean coastal database for assessing the impacts of sea-level rise and associated hazards. *Sci. Data* 5:180044 doi: 10.1038/sdata.2018.44 (2018).

**Publisher’s note:** Springer Nature remains neutral with regard to jurisdictional claims in published maps and institutional affiliations.

## Supplementary Material



## Figures and Tables

**Table 1:** Summary of the parameter and data included in the Mediterranean coastal database on a segment level.

**Parameter**	**Label**	**Description**	**Data source**	**Code**
Country code	ISO	ISO alpha-3 codes, Standard country codes for statistical use	International Organization for Standardization^41^	
Administrative boundaries	country_name	Country borders	GADM database of Global Administrative Areas^21^, version 2.8	
	admin_name	Administrative borders and their associated names	GADM database of Global Administrative Areas^21^, level 02, version 2.8	
	admin_id	Administrative unique identifier code associating each segment/assessment unit with the corresponding administrative information	-	
Distribution of assets and people	urban	Distinguishes the coast into two classes, (1) urban and (0) rural.	Google Earth imagery and location-tagged photographs from the web-service Panoramio; MODIS 500-m global map of urban extent dataset^22^	
Coastal material	Coast_material	Four different coastal material classes, namely (1) sand, (2) unerodible, (3) mud and (4) rock with pocket beaches have been classified.	Google Earth imagery and location-tagged photographs from the web-service Panoramio	
Coastal length	length	Coastline segment length [in km], the Equidistant conic (world) projection was used.	-	
Latitude	lati	Latitude of the midpoint of the coastline segment [in decimal degrees]	-	
Longitude	longi	Longitude of the midpoint of the coastline segment [in decimal degrees]	-	
Segment identifier code	locationid	Unique numerical identifier code which links each coastline segment to the attributed data	-	
Coastal assessment unit	coastalass_unit	Indicates the coastal assessment unit that belongs to the segment and lies directly at the coast. (Segments that are smaller than the resolution of the assessment units (~ 90m) do not have an assessment unit. Those coastal assessment units are indicated with 999 as the first three digits)	-	
GDP	gdpc_year	GDP per capita in current international $ for 1995, 2000, 2005, 2010, 2015 (For countries were records are missing we used the values from GBR for GIB, FRA for MCO; for LYB and MNE we used the gdpc growth rates from IIASA to calculate 1995, There is no data for SYR (all years) and LBY (2015) provided by the World bank)	World Bank^42^	
GDP growth rates	gdpcGRSSPx_year	Annual average growth rate per capita [%] for every SSP on a country level from 2000 -2100 (5yr time steps). For small countries, no SSP data exists. Therefore, we used for GIB the values of GBR, MCO – FRA and PSE – ISR.	International Institute for Applied Systems Analysis (IIASA) - SSP Database^43,44^	
Population growth rates	popGRSSPx_year	Annual average population growth rate [%] for every SSP on a country level from 2000 -2100 (5yr time steps). For small countries, no SSP data exists. We used for GIB the values of GBR, MCO – FRA and PSE – ISR.	International Institute for Applied Systems Analysis (IIASA) - SSP Database^43,45^	
River mouth	river	Includes the main estuaries and deltas of the Mediterranean, Non-river segment (0), river segment (1)	Derived from google earth	
	river_name	Name of the river mouth		
Extreme sea levels	GTSR_rp1 (2,5, 10,25, 50, 100, 250, 500, 1000)	1 in 1, 1 in 2, 1 in 5, 1 in 10, 1 in 25, 1 in 50, 1 in 100, 1 in 250, 1 in 500, 1 in 1000 year surge height (in base year) respectively, Height above mean sea level, [in m]	Based on the global reanalysis of storm surges and extreme sea level (GTSR) dataset^30^	surges.py
	DCESL _rp1 (10,100,1000)	1 in 1, 1in 10, 1in 100, 1 in 1000 year surge height (in base year) respectively, Height above mean sea level, [in m]	Vafeidis *et al*. 2008^46^	surges.py
Waves	waves	Mean wave height [in cm]	Dataset produced as part of the RISES-AM project by CMCC (Euro-Mediterranean Center on Climate Change)^4,34^	waves.py
Coastal Slope	cst	Topographic coastal slope [in degrees] derived from GEBCO [30 arc-seconds resolution]	GEBCO^47^	cst.py
Tide	maxhw	Max High Water [in m]	Pickering^48,49^	tide.py
	mhw	High Water [in m]	Pickering^48,49^	tide.py
	minlw	Minimum Low Water [in m]	Pickering^48,49^	tide.py
	mlw	Low Water [in m]	Pickering^48,49^	tide.py
	mtr	Mean Tidal Range [in m]	Pickering^48,49^	tide.py
Vertical land movement	VerticalMovement04	Average uplift/subsidence [in mm/yr] along the segment from estimates of glacio-isostatic adjustment.	Peltier (2004)^50^	uplift2004.py
	VerticalMovement14	Average uplift/subsidence [in mm/yr] along the segment from estimates of glacio-isostatic adjustment.	Peltier (2014)^51,52^	uplift2014.py
Saltmarshes	saltmarshes	Area of salt marsh within a coastal segment [in km^2^]	UNEP-WCMC^53^	wetland.py
Sea-level rise	RCP26(45,85)_1995(-2100)_hig	Regionalized SLR scenarios, which account for regional gravitational and rotational effects due to changes in ice mass distribution and steric changes. Mean sea-level rise relative to 1985-2005 [m] for RCP 2.6, 4.5 and 8.5 for a high ice-sheet melting scenario.	Hinkel *et al*., 2014^13^	SLR_hig.py
	RCP26(45,85)_1995(-2100)_med	Regionalized SLR scenarios, which account for regional gravitational and rotational effects due to changes in ice mass distribution and steric changes. Mean sea-level rise relative to 1985-2005 [m] for RCP 2.6, 4.5 and 8.5 for a medium ice-sheet melting scenario.	Hinkel *et al*., 2014^13^	SLR_med.py
Tourist arrivals	Tour_arryear	International tourism, number of arrivals on a country level form 1995-2014, 0 equals NoData	World Bank^54^	
Mean dynamic ocean topography	MDT	The MDT is the difference between the mean sea surface and the geoid over the 1993-2012 period [in m]. This parameter can be used to correct the offset for instance between extreme water levels and elevation data^31^	Rio *et al*.^55^	MDT.py

**Table 2:** Summary of the parameter and data included in the Mediterranean coastal database on at coastal assessment unit level.

**Parameter**	**Label**	**Description**	**Data source**	**Code**
Area below 1…20 m	area1…20	Hydrological connected area [in km^2^] at elevation increment x, corresponding to every coastal assessment unit. In order to calculate the earth surface area we generated a ‘real’ area grid based on the spheroidal approximation of the Earth surface^57^ and overlaid it with the SRTM data.	Shuttle Radar Terrain Mission (SRTM) data, 90m resolution^56^	area.py
Population living in area 1…20 m	popx_gpw2000	Total population living in elevation increment x based on the gridded population of the world, version 4 (ref. 59). UN-adjusted population estimates for 2000. The resolution is 30 arc-seconds, or ≈1 km at the equator.	Center for International Earth Science, Information Network^58^, Columbia University^59^	gpw00.py
	popx_gpw2010	Total population living in elevation increment x based on the gridded population of the world, version 4 (ref. 59). UN-adjusted population estimates for 2010. The resolution is 30 arc-seconds, or ≈1 km at the equator.	Center for International Earth Science, Information Network^58^, Columbia University^58^	gpw10.py
	popx_gr2000	Total population living in elevation increment x based on the Global Rural-Urban Mapping Project, Version1 (GRUMPv1). The resolution is 30 arc-seconds, or ≈1 km at the equator.	Center for International Earth Science Information Network^58^, Columbia University^58^	gr00.py
Landuse	ForestArea (UrbanArea, ArableArea, OpenArea)	The Globcover data includes 22 land cover classes defined by the United Nations (UN) Land Cover Classification System (LCCS). We reclassified this classification to 4 classes, namely forest, urban, arable land and open space. For every assessment unit the area for every class is calculated [in km^2^].	European Space Agency & Université Catholique De Louvain (UCL). Global Land Cover Map for 2009 (ref. 60)	landuse.py
	PC_forest (PC_Urban, PC_arable, PC_open)	Percent of every assessment unit that is covered by one of the landuse classes.	European Space Agency & Université Catholique De Louvain (UCL). Global Land Cover Map for 2009 (ref. 60)	landuse.py
Coastal parameter	coastal_zone	Indicates if a coastal assessment unit is lying directly at the coast (true) or inland (false)	-	
SSP	GR_SSP1(-5)_2010(-2100)_med	Spatial Population growth rates for the Mediterranean Coastal Zone per coastal assessment unit and SSP. These are based on Regionalized Shared Socioeconomic Pathways which are based on GPWv4 (ref. 59).	Reimann *et al*.^61^	SSPs_med.py
	GR_SSP1(-5)_2010(-2100)_global	Global spatial Population growth rates per coastal assessment unit and SSP. These are based on global gridded population projections for the coastal zone under the Shared Socioeconomic Pathways. Based on GRUMP 2000 (ref. 58).	Merkens *et al*.^62^	SSP_global.py
	SSP1(-5)_2010(-2100)_med	Total population numbers generated for every time step from Regionalized Shared Socioeconomic Pathways. Based on GPWv4 (ref. 59)	Reimann *et al*.^61^	SSPs_med.py
	SSP1(-5)_2010(-2100)_global	Total population numbers generated for every time step from global gridded population projections. Based on GRUMP 2000 (ref. 58).	Merkens *et al*.^62^	SSP_global.py

**Table 3:** Comparison of coastal morphology classification.

**MCD**	**Length [km]**	**Percent of total coastline length**	**MEDSEA**	**Length [km]**	**Percent of total coastline length**
(1) Sandy	12,493	23.0	(1) Sandy beach and beach with uncertain grain size	11,929	23.8
(2) Unerodible	25,051	46.1	(2) River deltas, estuaries and soft sedimentary strands	1,802	3.6
(3) Muddy	3,570	6.6	(3) Artificial structures and artificial frontage	4,936	9.8
(4) Rocky with pocket beaches	13,181	24.3	(4) Soft Rock shores	16,163	32.2
			(5) Hard Rock shores	15,322	30.5
Total coastline length	54,296	100	Total coastline length	50,153	100
